# Association of Differing Qatari Genotypes with Vitamin D Metabolites

**DOI:** 10.1155/2020/7831590

**Published:** 2020-04-13

**Authors:** Youssra Dakroury, Alexandra E. Butler, Soha R. Dargham, Aishah Latif, Amal Robay, Ronald G. Crystal, Stephen L. Atkin

**Affiliations:** ^1^Weill Cornell Medicine-Qatar, P.O. Box 24144, Doha, Qatar; ^2^Diabetes Research Center (DRC), Qatar Biomedical Research Institute (QBRI), Hamad Bin Khalifa University (HBKU), Qatar Foundation (QF), Doha, Qatar; ^3^Antidoping Laboratory, Doha, Qatar; ^4^Department of Genetic Medicine, Weill Cornell Medicine, New York, USA; ^5^Royal College of Surgeons Ireland, Busaiteen, Bahrain

## Abstract

**Objective:**

Genetic studies have identified four Qatari genotypes: Q1 Arab, Bedouin; Q2 Asian/Persian; Q3 African; and a fourth admixed group not fitting into the previous 3 groups. This study was undertaken to determine if there was an increased risk of deficiency of vitamin D and its metabolites associated with differing genotypes, perhaps due to genetic differences in skin pigmentation.

**Methods:**

398 Qatari subjects (220 type 2 diabetes and 178 controls) had their genotype determined by Affymetrix 500 k SNP arrays. Total values of 1,25-dihydroxyvitamin D (1,25(OH)2D), 25-hydroxyvitamin D (25(OH)D), 24,25-dihydroxyvitamin D (24,25(OH)2D), and 25-hydroxy-3epi-vitamin D (3epi-25(OH)D) concentrations were measured by the LC-MS/MS analysis.

**Results:**

The distribution was as follows: 164 (41.2%) genotyped Q1, 149 (37.4%) genotyped Q2, 31 (7.8%) genotyped Q3, and 54 (13.6%) genotyped “admixed.” Median levels of 25(OH)D and 3epi-25(OH)D did not differ across Q1, Q2, Q3, and “admixed” genotypes, respectively. 1,25(OH)2D levels were lower (*p* < 0.04) between Q2 and the admixed groups, and 24,25(OH)2D levels were lower (*p* < 0.05) between Q1 and the admixed groups. Vitamin D metabolite levels were lower in females for 25(OH)D, 1,25(OH)2D (*p* < 0.001), and 24,25(OH)2D (*p* < 0.006), but 3epi-25(OH)D did not differ (*p* < 0.26). Diabetes prevalence was not different between genotypes. Total 1,25(OH)2D (*p* < 0.001), total 24,25(OH)2D (*p* < 0.001), and total 3epi-25(OH)D (*p* < 0.005) were all significantly lower in diabetes patients compared to controls whilst the total 25(OH)D was higher in diabetes than controls (*p* < 0.001).

**Conclusion:**

Whilst 25(OH)D levels did not differ between genotype groups, 1,25(OH)2D and 24,25(OH)2D were lower in the admixed group, suggesting that there are genetic differences in vitamin D metabolism that may be of importance in a population that may allow a more targeted approach to vitamin D replacement. This may be of specific importance in vitamin D replacement strategies with the Q2 genotype requiring less, and the other genotypes requiring more to increase 1,25(OH)2D. Whilst overall the group was vitamin D deficient, total 25(OH)D was higher in diabetes, but 1,25(OH)2D, 24,25(OH)2D, and 3epi-25(OH)D were lower in diabetes that did not affect the relationship to genotype.

## 1. Introduction

Next generation exome sequencing has identified three major genetic subgroups within the Qatari population (Q1 Bedouin, Q2 Persian-South Asian, and Q3 African) and has identified variants within genes that have effects on clinically significant Mendelian diseases [1–3]. Several of these Mendelian variants were only segregating in one Qatari subpopulation [2]. However, because of their initial origin, this may lead to differences in skin pigmentation that would potentially lead to differences in vitamin D akin to those with darker skins becoming vitamin D deficient in the northern hemisphere [4]. This, in turn, may exacerbate the already prevalent vitamin D deficiency seen in the Middle East and, in particular, in Qatar.

Vitamin D deficiency has become the most common nutritional deficiency throughout the world [5]. While vitamin D deficiency is a global issue [6], cultural norms dictating full body coverage in parts of the world such as the Middle East magnify the issue of vitamin D deficiency in these regions [7, 8]. Vitamin D is key in maintaining skeletal health, but it has been found to have a wider role in, for example, supporting cardiovascular health, and it may exert anticancer effects [9]. Tissue specific production of 1,25(OH)_2_D from its precursor 25(OH)D is essential for proper functioning of both the adaptive and the innate immune systems [10]. Vitamin D sufficiency is important for cellular and tissue homeostasis, and it has been shown to be associated with reduced all-cause mortality [11]. Obesity, which is a particular issue in the Middle East, can lead to decreased bioavailability of vitamin D and, therefore, deficiency because of deposition in the body fat compartments [12].

Vitamin D3 (cholecalciferol) is endogenously produced whilst vitamin D2 is derived from the diet as ergosterol, primarily from mushrooms and fungi, which is then converted to ergocalciferol by UV-B; both are hydroxylated to vitamin D2 (25(OH)D_2_) or vitamin D3 (25(OH)D_3_) by multiple 25-hydroxylases [13, 14] in the liver. Vitamin D is transported to the kidney and converted either to the active 1,25(OH)_2_D by 1 alpha hydroxylase or to 24,25(OH)_2_D [15].

It has been recently reported that extrarenal tissues may also convert 25(OH)D to 1,25(OH)_2_D [16]. 1,25(OH)_2_D binds to the vitamin D receptor (VDR) and subsequently heterodimerizes with the retinoid X receptor for its action [15]. Vitamin D2 is available both as a supplement and as a pharmaceutical to treat vitamin D deficiency.

3epi-25(OH)D is formed by 3-epimerase [13, 17] and is relatively inactive compared with 25(OH)D, the main issue being that it may be measured inadvertently whilst measuring 25(OH)D, leading to 25(OH)D overestimation [18]. However, the 3-epimer may be as potent as 1,25(OH)_2_D_3_ on PTH suppression [13, 19], but there is less data on any other biological effects it may possess.

Epidemiological evidence has related vitamin D deficiency to an increased risk of type 2 diabetes (T2DM) [20, 21], with vitamin D deficiency associated with both insulin resistance and beta cell dysfunction [22], contributing to metabolic syndrome and T2DM [23, 24]. Vitamin D has been associated with poorer glycemic control and increased mortality [25], and given the marked vitamin D deficiency in this population [26], this may lead to increased microvascular complications of retinopathy, neuropathy, and nephropathy [27, 28].

We hypothesized that the skin pigmentation associated with the differing Qatari genotypes would have an impact on vitamin D and its metabolites, perhaps exacerbated by the presence of diabetes, which may have major implications on vitamin D replacement strategies, and therefore, this study was undertaken.

## 2. Methods

### 2.1. Study Population

The recruitment strategy for the subjects in this study has been described before [3]. Briefly, all subjects were over the age of 30, and there were a minimum of three generations Qatari. Cases were excluded if any of the following were present: history of type 1 diabetes, maturity onset diabetes of the young (MODY), maternally inherited diabetes and deafness syndrome (MIDD), a first degree relative with type 1 diabetes, or secondary diabetes [3]. The diagnosis of type 2 diabetes was made according to the WHO guidelines [29]; for inclusion in the type 2 diabetes cohort, at least one of the following was required: fasting plasma glucose >7  mmol/l, HbA1c > 6.5%, or a diagnostic glucose tolerance test. Inclusion in the nondiabetic control group required a normal glucose tolerance test. 398 subjects with no known familial relationships and satisfying the Qatari ancestry criteria and with unambiguous assignment to a Qatari subpopulation were selected from a group visiting the health clinics at Hamad Hospital, Doha, Qatar, for a routine diabetes screening [2]. Nondiabetic subjects were comprised of relatives accompanying the type 2 diabetic (T2DM) subjects. A total of 398 subjects were genotyped (Table 1).

Diabetes patients were on hypoglycemic therapy, and all subjects had been prescribed vitamin D_2_ supplements 50,000 units weekly.

The study was approved by Weill Cornell IRB (IRB# 13-00063), and all participants provided written informed consent. The conduct of the trial was in accordance with ICH GCP and the Declaration of Helsinki.

### 2.2. Study Design

Following an overnight fast, blood samples were collected, and weight and blood pressure were measured at the baseline visit. The fasting venous blood was collected into fluoride oxalate and serum gel tubes. Samples were separated by centrifugation at 2000 ×g for 15  minutes at 4°C, and the aliquots were stored at −80°C within 1 hour of collection. Overnight urine samples were collected, and aliquots were stored at −80°C until batch analysis. Blood pressure was measured using an automated device (NPB-3900; Nellcor Puritan Bennett, Pleasanton, CA) during each study visit. Blood pressure measurements were performed after the subjects had been seated quietly for at least 5 minutes and with the right arm supported at heart level. Three readings were taken, each at least 2 minutes apart, and then the average of the readings was obtained.

### 2.3. Serum Vitamin D Measurement

Serum vitamin D levels were quantified using isotope-dilution liquid chromatography tandem mass spectrometry (LC-MS/MS). 25 *μ*L of internal standards (d6-1calcitriol (1.5  ng/mL), d6-25OHD_3_ (50 ng/mL), and d6-25OHD_2_ (20 ng/mL) were added into each microcentrifuge tube containing 250  *μ*L of calibration standards, quality control, or serum samples and kept for 30 minutes to reach binding equilibrium. The samples were diluted with 250  *μ*L of pretreatment solution (isopropanol and water; 50 : 50 v/v) and left to stand for at least 15  min to displace binding protein.

300  *μ*L of pretreated samples were loaded onto ISOLUTE® supported liquid extraction (SLE+) columns (Biotage), followed by elution with 1.8  mL of n-heptane (2 × 900  *μ*L) into a collection tube already containing 200  *μ*L of 0.25  mg/mL PTAD solution in ethyl acetate and heptane (8 : 92 v/v). The eluate was evaporated to dryness using turbovap under nitrogen gas heated at 38°C. Once dried, 50  *μ*L of reconstituted solution consisting of methanol and deionized water, 70 : 30 v/v, and 0.006% methylamine were added into all tubes. The derivatised extracts were transferred into LC insert vials and 10  *μ*L from each was injected into the LC-MS/MS system. Vitamin D deficiency was defined according to the Endocrine Society: deficiency, insufficiency, and repletion as ≤ 20 ng/mL, 20–30  ng/mL, and ≥30  ng/mL, respectively.

### 2.4. Qatari Genetic Subpopulation Genotyping

DNA was extracted from blood using the QIAamp DNA Blood Maxi Kit (Qiagen Sciences Inc, Germantown, MD). The 398 subjects were classified into the three genetic subgroups described in the Qatari population [8, 9] using a TaqMan SNP Genotyping Assay (Life Technologies, Carlsbad, CA) for a previously described panel of 48 ancestry informative SNPs [8, 9]. Average genotype call rate was 96% and analyzed in STRUCTURE with *K* = 3. Q1, Q2, or Q3 population was assigned if the highest proportion was >65%; otherwise, they were classed as “admixed”.

### 2.5. Study Outcomes

#### 2.5.1. Statistical Analyses

Data trends were visually and statistically evaluated for normality. Nonparametric tests (Mann–Whitney U and Kruskall–Wallis tests) were applied on data that violated the assumptions of normality when tested using the Kolmogorov–Smirnov test. Bonferroni correction was applied to account for multiple testing. Statistical analysis was performed using SPSS for Windows, version 24.0. All values are given as mean ± SD or as mean with 95% confidence interval (CI) unless otherwise specified.

## 3. Results

### 3.1. Baseline Characteristics

The baseline characteristics for the entire cohort and those genotyped are shown in Table 1. It can be seen that there were no differences in age, gender, or BMI between the differing genotypes; however, the Q3 genotype had significantly more hypertension in the diabetes population (*p* < 0.04). Vitamin D status between males and females is shown in supplementary Table 1: 25(OH)D, 1,25(OH)2D (*p* < 0.001) and 24,25(OH)2D (*p* < 0.006) were significantly lower in females compared to males, but 3epi-25(OH)D did not differ (*p* < 0.26).

### 3.2. Vitamin D in relation to Qatari Genotype

The relationship of the Qatari genotypes to diabetes and vitamin D metabolites are shown in Table 2. Vitamin D metabolites between the diabetes and control subjects are shown in Table 3.

In the 398 subjects, the mean age was 49.8 years and 56.8% were males (Table 1). The distribution was as follows: 164 (41.2%) genotype Q1, 149 (37.4%) genotype Q2, 31 (7.8%) genotype Q3, and 54 (13.6%) genotyped “admixed”. Median levels of 25(OH)D,_,_ 1,25(OH)_2_D, and 3epi-25(OH)D did not differ across Q1, Q2, Q3, and “admixed” genotypes, respectively. 1,25(OH)_2_D levels significantly differed between Q2 and the admixed groups (*p* < 0.04), and 24,25(OH)_2_D significantly differed between Q1 and the admixed groups (*p* < 0.05) (Table 2).

Vitamin D metabolites and the relation to genotype was determined for the entire group that included subjects with and without diabetes, but as there was no difference in the association of diabetes with genotype, then this was a valid approach. The vitamin D levels in control and diabetes subjects are shown in Table 3. Overall the control group was vitamin D deficient (in accord with the Endocrine Society definition) with the median total 25(OH)D being 19.58 ng/ml (59.32) and the diabetes group vitamin D insufficient 26.46  ng/ml (17.84): total 25(OH)D was higher in diabetes (*p* < 0.001), but 1,25(OH)_2_D (*p* < 0.001), 24,25(OH)_2_D (*p* < 0.001), and 3epi-25(OH)D (*p*=0.005) were lower in diabetes (Table 3).

## 4. Discussion

This study clearly showed that the differing Qatari genotypes did not contribute to the levels of measurable 25(OH)D and each was as equally vitamin D deficient as the other. On one hand, this is surprising given the differing degrees of skin pigmentation [4]. Studies on Qatari genotype revealed 3 genotypes: Q1 Arab, Bedouin; Q2 Asian/Persian; and Q3 African, with Q3 likely having the most pigmented skin [1]. However, given the whole body coverage that is prevalent in the Middle East [7], then that is likely to be the prevailing and dominant factor in this instance rather than skin pigmentation. Vitamin D in the form of 25(OH)D is measured to ascertain vitamin D sufficiency [30] with the other metabolites being largely measured for research purposes [30]. In this case, 1,25(OH)2D levels significantly differed between Q2 and the admixed groups, perhaps through increased renal or extrarenal CYP27B1 activity, whilst 24,25(OH)2D significantly differed between Q1 and the admixed groups, perhaps through modulation of CYP27A1 activity. Thus, this suggests that there are potential genetic differences in vitamin D metabolism that would account for these findings including VDR gene polymorphisms [31], which may have impact on vitamin D supplementation in the deficiency state where higher supplementation may be required depending on the genotype. Hypertension was found to be significantly higher in the Q3 population, and it is recognized that vitamin D deficiency is associated with hypertension and repletion has an antihypertensive effect [32]; hypothetically, the Q3 genotype may be more sensitive to vitamin D depletion, but vitamin D metabolite levels were not associated with blood pressure.

Diabetes did not associate with any specific genotype, though the study population was too small to answer this question. None the less, as there was no association and the percentage of subjects with diabetes did not differ significantly between the genotypes, all subjects for any given genotype were grouped and compared with the vitamin D levels. Others have looked at the haplotype analysis showing that the Qatari haplotypes in the region of known diabetes risk in the Q1 and Q2 populations did not differ from European haplotypes [3], though exome sequencing has shown risk variants for Mendelian disorders at high prevalence in Qatar due to consanguinity [2]. Males showed higher levels of 25(OH)D, 1,25(OH)2D (*p* < 0.001), and 24,25(OH)2D, but not of 3epi-25(OH)D. Lower vitamin D concentrations in the Qatari females versus males are also in accord with other studies [33], though this is not a universal finding [27]. However, it should be noted that whilst overall the group was vitamin D deficient, when there was a comparison between controls and diabetes subjects, the total 25(OH)D was higher in diabetes, but 1,25(OH)_2_D, 24,25(OH)_2_D, and 3epi-25(OH)D were lower in diabetes. The levels of vitamin D_2_ were higher in diabetes, potentially reflecting the ingestion of vitamin D supplements by these subjects, though all subjects were being prescribed vitamin D2.

The role of vitamin D deficiency in the development of type 2 diabetes has been suggested [34–36], with epidemiological evidence linking vitamin D deficiency and insulin resistance [37, 38]. However, long-term studies have found no protective effect of vitamin D and calcium supplementation on the risk of diabetes [39], and supplementation in vitamin D replete T2DM patients did not affect insulin resistance or glycemic control [40].

A strength of the study is the measurement of the vitamin D metabolites by state of the art methods. It is well recognized that lifestyle and diet are important for vitamin D levels, and whilst all of the subjects were more than 3 generation Qataris who all followed whole body coverage and the Qatari lifestyle, a limitation of the study was that formal dietary and lifestyle assessment was not undertaken. Other limitations include the cross sectional nature of the study, and it is unknown if the vitamin D range for each metabolite was the same in each genotype though the interquartile ranges for each were no different for each metabolite. An additional limitation was that the estimated glomerular filtration rate was not measured that could have impacted on vitamin D metabolite levels.

In conclusion, 25(OH)D levels did not differ between groups, suggesting that skin coloration may not contribute to vitamin D status; however, the 1,25(OH)_2_D form was higher for Q2 and 24,25(OH)_2_D was higher in Q1 compared to the “admixed” group, suggesting that there are genetic differences in vitamin D metabolism that may be of importance in this population particularly if increased supplementation is needed for differing genotypes.

## Figures and Tables

**Table 1:** Demographics of the 398 subjects.

	Population
Age, mean (SD)	49.8 (10.6)
Gender, *N* (%)	
Male	226 (56.8)
Female	172 (43.2)
HBA1C, median (IQR)	6.9 (2.7)
Glucose, median (IQR)	6.1 (3.9)
Diabetes-yes, *N* (%)	220 (55.3)
Hypertension-yes, *N* (%)	186 (46.7)
Dyslipidemia-yes, *N* (%)	224 (56.3)

**Table 2:** Demographic data, genotype (Q1, Q2, Q3, and admixed), and vitamin D metabolites in 398 Qatari subjects.

	Q1	Q2	Q3	Admixed	*p* value
	Median (IQR)	Median (IQR)	Median (IQR)	Median (IQR)	
Gender *N* (%)					
Male	85 (51.8)	98 (65.8)	12 (38.7)	31 (57.4)	
Female	79 (48.2)	51 (34.2)	19 (61.3)	23 (42.6)	

Age, mean (SD)	49.5(11.1)	49.7 (10.0)	51.8 (9.6)	49.6 (11.2)	0.74

BMI	31.78 (28.62–36.30)	31.23 (27.69–35.35)	31.63 (29.39–36.12)	31.28 (28.49–36.61)	0.57

Diabetes, *N* (%)					
No	65 (39.6)	75 (50.3)	12 (38.7)	26 (48.1)	0.23
Yes	99 (60.4)	74 (49.7)	19 (61.3)	28 (51.9)	0.11

Hypertension *N* (%)					
No	87 (53.0)	86 (57.7)	9 (29.0)	30 (55.6)	0.04
Yes	77 (47.0)	63 (42.3)	22 (71.0)	54 (44.4)	0.04

HBA1C (%)	7.10 (5.95–8.55)	6.65 (5.70–8.60)	7.70 (6.43–8.98)	6.70 (5.80–8.95)	0.53

Glucose (mmol/l)	6.85 (5.28–9.30)	6.10 (5.00–9.00)	6.05 (5.15–9.55)	5.50 (4.90–8.60)	0.10

Total 25(OH)D (ng/ml), median (IQR)	22.76 (15.16–34.32)	20.77 (14.61–31.09)	22.27 (13.03–30.16)	22.98 (13.65–34.23)	0.39

Total 1,25(OH)_2_D (ng/ml), median (IQR)	0.034 (0.018–0.053)	0.042 (0.022–0.065)	0.038 (0.011–0.054)	0.025 (0.015–0.048)	0.04

Total 3epi-25(OH)D (ng/ml), median (IQR)	0.33 (00.20–0.79)	0.55 (0.0.22–0.90)	0.40 (0.14–1.16)	0.36 (0.21–0.55)	0.10

Total 24,25(OH)_2_D (ng/ml), median (IQR)	0.36 (0.22–0.62)	0.34 (0.19–0.63)	0.30 (0.18–0.42)	0.25 (0.19–0.44)	<0.05

HbA1c = glycated hemoglobin A1c; 25(OH)D = 25-hydroxyvitamin D; 1,25(OH)_2_D = 1,25-dihydroxyvitamin D; 3epi-25(OH)D = 25-hydroxy-3epi-vitamin D (3epi-25(OH)D); 24,25(OH)_2_D = 24, 25-dihydroxyvitamin D.

**Table 3:** Vitamin D levels between diabetes (*n* = 220) and controls (*n* = 178).

	Control	Diabetes	*p* value
	Median (range)	Median (range)	
Age (years)	46.1 (10.8)	55.2 (9.9)	<0.001
BMI (kg/m^2^)	30.1 (34.8)	32.4 (44.0)	<0.001
HbA1c (%)	5.6 (4.6)	7.9 (11.2)	<0.001
Glucose (mmol/L)	5.2 (14.6)	8.6 (26.7)	<0.001
Total 1,25(OH)_2_D (ng/ml)	0.04 (2.08)	0.02 (0.19)	<0.001
Total 25(OH)D (ng/ml)	19.6 (59.3)	26.5 (17.8)	<0.001
Total 24,25(OH)_2_D (ng/ml)	0.387 (4.49)	0.290 (7.77)	<0.001
Total 3epi-25(OH)D (ng/ml)	0.387 (4.49)	0.290 (7.77)	0.005

BMI = body mass index; HbA1c = glycated hemoglobin A1c; 1,25(OH)_2_D = 1,25-dihydroxyvitamin D; 25(OH)D = 25-hydroxyvitamin D; 24,25(OH)_2_D = 24, 25-dihydroxyvitamin D; 3epi-25(OH)D = 25-hydroxy-3epi-vitamin D (3epi-25(OH)D).

## Data Availability

All data relating to this study will be made available by the corresponding author upon reasonable request.

## Supplementary Materials

Supplementary Materials Supplementary Table 1: vitamin D metabolites according to gender.
