# Supplementary material for: Association of Differing Qatari Genotypes with Vitamin D Metabolites
Source: Int J Endocrinol. 2020 Apr 13;2020:7831590. doi: 10.1155/2020/7831590 (PMC7174927; doi:10.1155/2020/7831590)
Supplement: Supplementary Materials — Supplementary Table 1: vitamin D metabolites according to gender. [file 7831590.f1.pdf]

Supplementary Table 1. Vitamin D metabolites according to gender

	Females	Males	Significance
	**Median (IQR)**	**Median (IQR)**	
**Total $1,25(OH)_2D$ (ng/ml)**	0.01 (0.00-0.04)	0.04 (0.02-0.06)	<0.001
**Total 25(OH)D (ng/ml)**	24.12 (18.83-36.88)	18.73 (13.12-29.58)	<0.001
**Total 3epi-25(OH)D (ng/ml)**	0.14 (0.00-0.36)	0.28 (0-0.78)	0.26
**Total $24,25(OH)_2D$ (ng/ml)**	0.24 (0.17-0.51)	0.39 (0.24-0.62)	0.006

$1,25(OH)_2D$ =1,25-dihydroxyvitamin D; 25(OH)D = 25-hydroxyvitamin D;
3epi25(OH)D = 25-hydroxy-3epi-Vitamin D (3epi25(OH)D); $24,25(OH)_2D$ =24, 25-dihydroxyvitamin D
